# Dry Reforming of Methane over Ni/WC Catalysts: Effect of Ni Content and CH_4_:CO_2_ Ratio

**DOI:** 10.3390/ma18173990

**Published:** 2025-08-26

**Authors:** Zhanar Bolatova, Svetlana Kuznetsova, Olga Vedishcheva, Sónia A. C. Carabineiro, Ekaterina Kolobova, Alexey Pestryakov

**Affiliations:** 1 School of Energy & Power Engineering, National Research Tomsk Polytechnic University, Lenin Av. 30, 634050 Tomsk, Russia; zsb3@tpu.ru; 2 Research School of Chemistry & Applied Biomedical Sciences, National Research Tomsk Polytechnic University, Lenin Av. 30, 634050 Tomsk, Russia; snk15@tpu.ru; 3 Laboratory of Radioecology and Marine Radiochemistry, Sevastopol State University, Universitetskaya st. 33, 299053 Sevastopol, Russia; ovvedisheva@mail.sevsu.ru; 4 LAQV-REQUIMTE, Department of Chemistry, NOVA School of Science and Technology, Universidade NOVA de Lisboa, 2829-516 Caparica, Portugal; sonia.carabineiro@fct.unl.pt

**Keywords:** CH_4_ dry reforming, tungsten carbide, Ni-based catalyst, catalyst stability, oxidation and coking resistance

## Abstract

Dry reforming of methane (DRM) into synthesis gas (CO + H_2_) is one of the most important chemical reactions for industrial hydrogen production. It also enables the synthesis of hydrocarbons (liquid fuels) and other valuable products, providing an effective route for utilizing greenhouse gases. However, a major challenge limiting the implementation and scale-up of DRM is the high cost of stable and active noble metal-based catalysts, or the rapid deactivation of nickel- and cobalt-based catalysts due to coking and sintering of the active metal particles. In this context, the present work demonstrates that combining a highly active and inexpensive component (Ni) with tungsten carbide produces a composite material exhibiting high catalytic activity and resistance to oxidation and coking during DRM. Tungsten carbide was synthesized using a vacuum-free electric arc method, and nickel was subsequently deposited in varying amounts (1–25 wt.%) using the deposition–precipitation method with NaOH (DP). The resulting catalysts were characterized by X-ray diffraction, temperature-programmed reduction and Raman spectroscopy. Their performance was evaluated under DRM conditions, at atmospheric pressure and 800 °C, using different CH_4_:CO_2_ ratios. The most effective oxidation/(re)carbonization cycle, ensuring catalyst stability during DRM by balancing the rates of carbon formation and removal from the catalyst surface, was achieved with a nickel content of 20 wt.% and a CH_4_ to CO_2_ ratio of 0.67 in the feed gas mixture.

## 1. Introduction

In today’s global society, growing attention is being paid to the challenge of global warming, with an increasing number of countries recognizing the urgent need to mitigate the escalating negative impacts of human activity on the environment, particularly the increasing emissions of greenhouse gases, such as carbon dioxide (CO_2_) and methane (CH_4_). According to the Intergovernmental Panel on Climate Change, continued inaction could result in a global temperature increase of 1.5 °C by 2030 and 2 °C by 2050, with further rises expected thereafter. Such temperature changes are projected to have catastrophic consequences, including unprecedented sea level rise, widespread flooding, forest fires, extreme weather events, famine and the destruction of wildlife habitats [1]. In this context, research into strategies and technologies for CO_2_ capture and storage (CCS) has intensified. At the same time, the scientific community has increasingly begun to view CO_2_ not merely as a costly waste product but as a valuable C_1_ building block for the production of fuels and high-value chemicals. Among the promising approaches for the utilization of CO_2_, alongside CH_4_ or associated petroleum gas, is their catalytic conversion into desirable products, such as hydrogen and synthesis gas [2,3].

Dry reforming of methane (DRM) (Equation (1)) is considered a particularly attractive route for producing synthesis gas or hydrogen, which are key feedstocks for numerous large-scale chemical processes [4,5]. A major advantage of DRM is its simultaneous utilization of two potent greenhouse gases (CO_2_ and CH_4_), making it environmentally beneficial. However, the lack of a catalyst that is both highly active and stable under reaction conditions remains a significant challenge.

CH_4_ + CO_2_ → 2CO + 2H_2_ (1)

Recent research has focused on developing metal-modified heterogeneous catalysts for DRM. Group VIII noble metals exhibit the highest catalytic activity and are significantly less susceptible to coking, offering superior stability under reaction conditions [6,7,8]. Nevertheless, their high cost and limited availability restrict large-scale industrial use. As an alternative to noble metal-based catalysts, supported Ni and Co catalysts have attracted considerable attention, as they can provide catalytic performance comparable to noble metals [9,10,11,12,13,14,15,16,17]. However, unlike noble metals, Ni- and Co-based catalysts often suffer from poor stability in DRM due to coke formation and sintering of active metal particles at high reaction temperatures. Another promising materials for DRM are metal carbides, particularly tungsten carbide (WC) and molybdenum carbide (Mo_2_C) [18,19,20,21,22,23,24,25,26,27]. These materials can offer good catalytic performance; however, their stability is generally maintained only at elevated pressures. Under atmospheric pressure, metal carbides are susceptible to oxidation during the reaction, forming inactive oxides and thereby losing effectiveness [28].

To address these limitations, recent studies have explored combinations of Ni or Co with metal carbides to exploit potential synergistic effects that enhance both catalytic activity and stability. Our previous work [29], along with studies by other authors [30,31,32,33,34,35,36,37,38,39,40,41,42], has demonstrated that coupling a highly active and inexpensive metal component (such as Ni or Co) with a metal carbide (e.g., WC, Mo_2_C) can yield catalysts with superior activity and greater resistance to oxidation and poisoning by carbon deposition during DRM, outperforming both nickel and cobalt-based catalysts supported on oxide carriers as well as the individual tungsten and molybdenum carbides. However, a key challenge that remains is ensuring the long-term stability of such catalysts under DRM conditions, particularly over extended time-on-stream (TOS) periods (≥80 h TOS). The present study aims to address this gap by optimizing the catalyst composition, identifying the most effective nickel loading, and evaluating the influence of the feed gas composition on catalytic activity and long-term stability during DRM.

The present study builds on these findings by systematically investigating the influence of Ni loading and CH_4_:CO_2_ feed ratio on Ni/WC catalysts, with a focus on activity, stability and coke resistance under prolonged operations. To the best of our knowledge, this is the first work to demonstrate effective coke suppression to ~1% over 200 h of continuous DRM by simultaneously optimizing catalyst composition and feed gas ratio. These results not only advance understanding of Ni–carbide systems but also provide practical guidelines for designing durable, high-performance DRM catalysts for sustainable CH_4_ and CO_2_ utilization.

## 2. Materials and Methods

### 2.1. Synthesis of the Support

Tungsten carbide was synthesized using a specialized laboratory electric arc setup. The main advantage of this system is its ability to create a self-shielding effect within the reaction zone during plasma treatment, effectively isolating it from atmospheric air. The setup consists of an inverter power supply connected to a graphite cathode (cup) and a graphite anode (rod). The anode is mounted on a linear electric drive with a stepper motor, enabling precise vertical movement above a stationary cathode. High-purity tungsten (Rare metals, Novosibirsk, Russia) and carbon (Promsnab, Tomsk, Russia) powders (≥99.9 wt.%) with an average particle size of 5–7 μm were used as the starting materials. The powder mixture was evenly spread across the bottom of the cathode and covered with a layer of graphite felt, which absorbed the arc discharge and stabilized the plasma zone. The synthesis parameters (operating current of 220 A, open-circuit voltage of 63 V and exposure time of 60 s) were selected based on previous studies [29,43], which confirmed the feasibility of tungsten carbide formation under these conditions.

### 2.2. Synthesis of Catalysts

**Figure 1:**
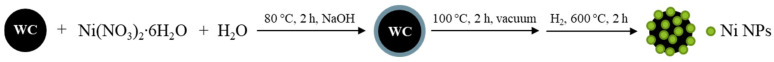
Schematic representation of the catalyst preparation process.

The catalysts were prepared by a deposition–precipitation (DP) method using NaOH. An aqueous solution of Ni(NO_3_)_2_∙6H_2_O (Sigma-Aldrich, St. Louis, MO, USA) was mixed with the support material, and the suspension was heated to 80 °C. A 0.5 M NaOH (Bashkir Soda Company JSC, Sterlitamak, Russia) solution was added dropwise until the pH increased from an initial value of 3 to 9. The mixture was maintained at 80 °C for 2 h under vigorous stirring to promote uniform precipitation and deposition of the nickel species onto the support. The resulting precipitates were repeatedly washed and centrifuged to remove residual ions, then dried under vacuum at 100 °C for 2 h. The synthesis procedure is illustrated in Figure 1 [29].

The nominal nickel content in the catalysts ranged from 1 to 25 wt%. After nickel deposition onto the support, the materials underwent drying, after which they were pre-treated in a H_2_ atmosphere at 600 °C for 2 h to reduce the metal species. The resulting as-synthesized catalysts were designated as X%Ni, where X denotes the nickel content in wt.%. The samples collected after DRM testing were labeled as X%Ni_SP, indicating spent catalysts.

### 2.3. Characterization of Support and Catalysts

The crystal phases of the catalysts were identified by X-ray diffraction (XRD) using a Shimadzu XRD 7000s diffractometer (SHIMADZU CORPORATION, Tokyo, Japan) with Cu Kα radiation (λ = 1.5406 Å). Measurements were performed at a tube voltage of 40.0 kV and a current of 30.0 mA, over an angular range of 2θ from 10 to 90 degrees. Qualitative phase analysis was carried out using the PDF4+ database, while quantitative analysis was performed using the corundum numbers (Reference Intensity Ratio, RIR) method and Rietveld method (PowderCell 2.4, 8.03.2000, W. Kraus & G. Nolze, Berlin, Germany).

The R_exp_ parameter, reflecting the quality of the XRD fitting ranged from 1.2 to 3.5% for the studied samples. The average crystallite size was estimated from the XRD peak broadening using the Debye–Scherrer equation (Origin PRO 2024, OriginLab Corporation, Northampton, MA, USA), applied to the main diffraction peaks of the identified phases.

Temperature-programmed reduction (TPR) measurements of both as-synthesized and spent catalysts were conducted using a Chemosorb chemisorption analyzer (NEOSIB, Novosibirsk, Russia) equipped with a thermal conductivity detector (TCD). Approximately 200 mg of each sample was placed into a U-shaped reactor. A gas mixture of 10% H_2_ in Ar was passed through the reactor at a flow rate of 20 mL/min. The temperature was increased from 30 °C to 800 °C at a heating rate of 10 °C/min. To prevent the influence of water vapor on the TPR signal, a cold trap containing a liquid nitrogen and isopropanol mixture was installed in the gas line.

Raman spectroscopy was performed on both as-synthesized and spent catalysts using a confocal Raman spectrometer (Confotec Uno, SOL instruments, Ltd., Minsk, Belarus) equipped with a 532 nm laser and a ×40 objective lens. Spectra were recorded in air at room temperature with spectral a resolution of 6.8–7.5 cm^−1^, a scanning range of 70–4740 cm^−1^ and an acquisition time of 5 s.

All measurements (XRD, H_2_–TPR and Raman spectroscopy) for each sample were performed at least twice to ensure reproducibility.

Further details of previous characterizations can be found in our previous work [29].

### 2.4. Catalytic Experiments

The catalytic performance of the synthesized materials was evaluated in DRM in a quartz flow reactor with a fixed catalyst bed. The reaction was conducted at 800 °C under atmospheric pressure and at a weight hourly space velocity (WHSV) of 12,000 mL∙g^−1^∙h^−1^. The composition of the feed gas mixture was varied by adjusting the CO_2_ content (50–70%) and CH_4_ content (30–50%).

Analytical monitoring of the catalytic process was carried out using a CHROMOS GC–1000 gas chromatograph (CHROMOS, Dzerzhinsk, Russia), connected to the reactor system via an online sampling line. The chromatograph was equipped with two TCDs and two separate packed columns: one filled with CaA sorbent (for the analysis of O_2_ and H_2_) and the other with AG–3 sorbent (for the analysis of CH_4_, CO_2_ and CO). Helium was used as the carrier gas. Quantification of the concentrations of reagents and reaction was performed using the absolute calibration method.

Catalyst performance was evaluated based on the conversions of CO_2_ and CH_4_ and the H_2_:CO product ratio. These parameters were calculated according to the following equations:

(2) $$X_{{\mathrm{CO}}_{2}}=\frac{{\left[{\mathrm{CO}}_{2}\right]}_{\mathrm{in}}-\;{\left[{\mathrm{CO}}_{2}\right]}_{\mathrm{out}}}{{{[\mathrm{CO}}_{2}]}_{\mathrm{in}}},$$

(3) $$X_{{\mathrm{CH}}_{4}}=\frac{{\left[{\mathrm{CH}}_{4}\right]}_{\mathrm{in}}-{\left[{\mathrm{CH}}_{4}\right]}_{\mathrm{out}}}{{{[\mathrm{CH}}_{4}]}_{\mathrm{in}}},$$

(4) $$\frac{H_{2}}{\mathrm{CO}}=\frac{{\left[H_{2}\right]}_{\mathrm{out}}}{{\left[\mathrm{CO}\right]}_{\mathrm{out}}},$$

where [i]_in_ and [i]_out_ are the concentrations of component i (CO_2_, CH_4_, H_2_ or CO) in the inlet and outlet gas streams, respectively.

For long-term TOS experiments, the first sample was taken 1 h after the temperature reached 800 °C, followed by additional measurements every 10 h. Regarding reproducibility, each gas chromatographic (GC) measurement was performed in duplicate, with the results showing excellent consistency (2%).

## 3. Results and Discussion

In our previous study [29], we investigated the nature of the active component(s) and the influence of catalyst preparation methods on catalytic performance in DRM, varying the reaction temperature (600–800°C) and WHSV (3600–12,000 mL∙g^−1^∙h^−1^). A series of nickel, cobalt and nickel–cobalt catalysts was prepared by incipient wetness impregnation and deposition–precipitation with NaOH, using tungsten carbide synthesized by a vacuum-free electric arc method as the support.

Comprehensive characterization revealed that the monometallic nickel catalyst prepared by deposition–precipitation with NaOH (designated as 20%Ni/WC_DP in [29]), was the most effective for DRM, owing its favorable structural, textural and electronic properties, as well as its resistance to oxidation and coking. This catalyst exhibited a specific surface area of 38.4 m^2^/g and consisted of 15.4 nm nickel nanoparticles supported on mixed tungsten carbide phases (WC and W_2_C).

After DRM, the phase composition shifted to predominantly nickel, with nearly complete conversion of W_2_C to WC, and the appearance of a graphite phase. During 80 h of TOS, the catalyst showed only modest deactivation, approximately 10% for CH_4_ conversion and 5% for CO_2_ conversion. We attributed this deactivation to a higher rate of carbon deposition compared to its removal by the reverse Boudouard reaction and/or the oxidation/(re)carbonization cycle. We further hypothesized that this effect could potentially be mitigated by increasing the CO_2_ concentration or modifying the catalyst composition.

Characterized by a structure with lattice distortions and stacking faults, the present study seeks to minimize catalyst deactivation by optimizing both the nickel loading and the reaction conditions, particularly the feed gas composition. For this purpose, a series of monometallic nickel catalysts with nickel contents varying from 1 to 25 wt.% was prepared by deposition–precipitation using NaOH. The textural, morphological, and compositional features of the Ni/WC catalysts studied here—determined by N_2_ adsorption–desorption, TEM, EDX and SAED—were comprehensively reported in our previous work [29], which employed the same preparation method and support material. Table 1 presents the main physicochemical parameters of the 20% Ni catalyst from that study, which are directly relevant to the interpretation of the current results. The catalysts were characterized by X-ray diffraction, H_2_–TPR and Raman spectroscopy before and after DRM. Their catalytic performance was systematically evaluated, with particular attention to the effect of feed gas composition and long-term stability over reaction times of up to 200 h.

### 3.1. XRD

**Figure 2:**
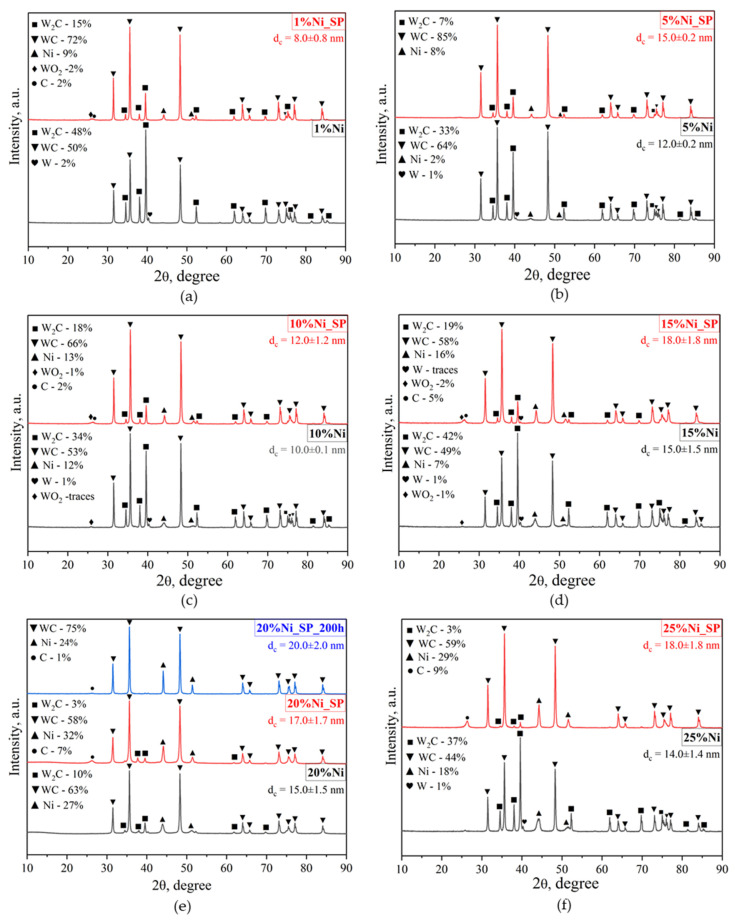
XRD patterns of as-synthesized and spent catalysts: (**a**) 1%Ni and 1%Ni_SP, (**b**) 5%Ni and 5%Ni_SP, (**c**) 10%Ni and 10%Ni_SP, (**d**) 15%Ni and 15%Ni_SP, (**e**) 20%Ni, 20%Ni_SP and 20%Ni_SP_200h, (**f**) 25%Ni and 25%Ni_SP. DRM conditions: T = 800 °C, P = 1 atm, CH_4_:CO_2_ ratio = 1.00, WHSV = 12,000 mL∙g^−1^∙h^−1^.

As demonstrated in our previous study [29], the support obtained by the vacuum-free electric arc treatment method comprises predominantly hexagonal phases of WC (79%, ICDD no. 00-025-1047) and W_2_C (21%, ICDD no. 00-035-0776). The as-synthesized catalysts also exhibit the presence of WC and W_2_C phases, with their contents varying between 44 and 64 wt.% and 10–48 wt.%, respectively (Figure 2). Additionally, a cubic tungsten phase (ICDD no. 01-089-2767) was detected in nearly all catalysts, present at approximately 2 wt.%. For samples containing 10 and 15 wt.% nickel, a monoclinic tungsten oxide (ICDD no. 01-071-0614) was observed in amounts up to 1 wt.% (Figure 2c,d). Beyond the support-related phases, diffraction peaks corresponding to cubic metallic Ni phase (ICDD no. 00-210-2284) were identified in almost all catalysts, with their intensity increasing with nickel loading (Figure 2). The only exception was the sample containing 1 wt.% Ni, in which the nickel peaks were below the XRD detection limit (Figure 2a).

The average nickel crystal size (d_c_) calculated from XRD for the as-synthesized samples ranged from 10 to 15 nm (Figure 2a–f). Samples containing 5 and 10 wt.% Ni exhibited smaller crystallite sizes (10–12 nm) compared to those with 15–25 wt.% Ni (14–15 nm). Notably, the average crystallite size determined from XRD for the 20 wt.% Ni sample (15 nm, Figure 2e) is in complete agreement with the TEM results reported in our previous study [29] (Table 1).

Figure 2 also presents the XRD results of the spent catalysts. Analysis of these patterns reveals phase transformations occurring during the DRM reaction, namely a partial transition of the W_2_C phase to WC and the disappearance of the metallic tungsten phase, likely due to carbonization. In addition, diffraction peaks corresponding to the hexagonal graphite phase (ICDD no. 00-058-1638) were observed for all spent catalysts, with their intensity increasing alongside nickel loading. For samples containing 1, 10 and 15 wt.% (Figure 2a,c,d), small reflections (≤2 wt.%) attributed to monoclinic tungsten oxide were also detected, indicating partial oxidation of tungsten carbide by CO_2_ during DRM. Importantly, no diffraction peaks corresponding to Ni oxides or nickel tungstates were observed in either the as-synthesized or spent catalysts. This indicates that, under the chosen preparation and reaction conditions, metallic Ni remains stable, which is consistent with our previous findings for nickel–cobalt and cobalt catalysts [29].

In addition to observed phase transformations, agglomeration of nickel nanoparticles was also detected in the spent catalysts (Figure 2a–f). The average Ni crystallite size increased by approximately 3 nm compared to the as-synthesized samples, ranging from 12 to 18 nm.

### 3.2. H_2_–TPR

**Figure 3:**
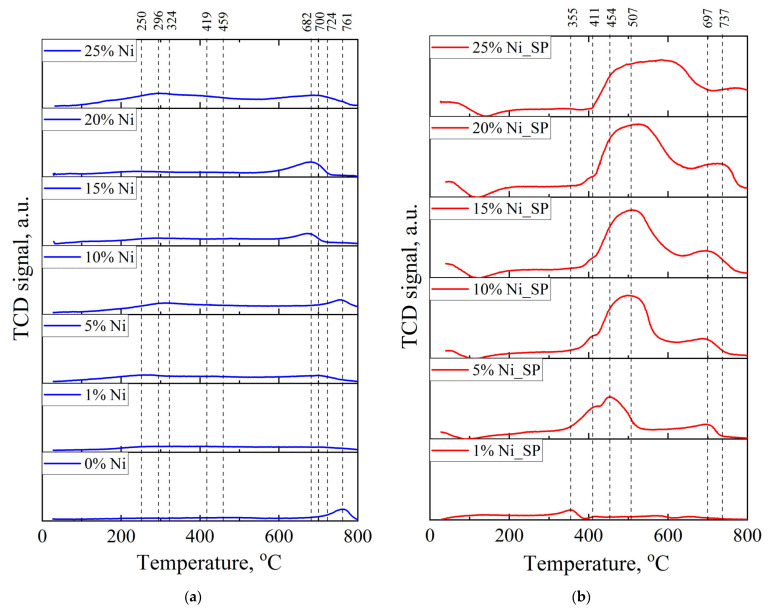
H_2_–TPR profiles of (**a**) as-synthesized and (**b**) spent catalysts. DRM conditions: T = 800 °C, P = 1 atm, CH_4_:CO_2_ ratio = 1.00, WHSV = 12,000 mL∙g^−1^∙h^−1^.

Figure 3 shows the H_2_–TPR profiles of the support, as-synthesized and spent catalysts. For the support (Figure 3a), a single hydrogen consumption peak is observed in the temperature range of 680–800 °C, corresponding to the reduction of tungsten oxides through the reactions WO_3_ + H_2_ → WO_2_ + H_2_O and WO_2_ + H_2_ → W + H_2_O [44]. For the as-synthesized catalysts with 1% and 5% Ni, only very weak hydrogen consumption is detected between 100 and 400 °C. This low-temperature peak is attributed to the reduction in highly dispersed oxidized nickel species and/or residual nickel precursor (like Ni(OH)_2_, which reduces around 200 °C) that was not completely reduced during the pre-treatment at 600 °C in H_2_ (Figure 3a). The reduction in adsorbed “atmospheric” impurities on the catalyst surface may also contribute to this signal [34,35,36,45,46]. As the nickel loading increases from 10 to 25%, the intensity of hydrogen consumption in the low-temperature region increases accordingly, which is likewise ascribed to the reduction in highly dispersed nickel oxide weakly interacting with the support, residual nickel precursors and/or surface impurities.

In addition, these catalysts exhibit extra reduction maxima at higher temperatures (550–750 °C), distinct from the support peak (680–800 °C). These high-temperature peaks likely correspond to the simultaneous reduction in oxidized nickel species and tungsten oxides, indicating a strong interaction between the active nickel component and the tungsten carbide support. It should be noted that XRD analysis (Figure 2) did not detect bulk nickel oxide or nickel tungstate phases in the as-synthesized catalysts, and only minor amounts of tungsten oxide (WO_2_) were found (≤1 wt.%). Therefore, the observed reduction peaks originate from surface oxide states rather than bulk oxide phases.

For the spent catalysts (Figure 3b), hydrogen consumption peaks in the low-temperature region (up to 400 °C) are practically absent, while a pronounced increase in hydrogen consumption is observed in the of 400–800 °C range. With increasing nickel content, the shape of hydrogen consumption profile changes, exhibiting peak broadening that likely reflects the overlap of multiple reduction and co-reduction processes involving both the active component and the support. For example, the catalyst with 5 wt.% Ni displays three distinct reduction peaks at 411, 454 and 697 °C in its TPR profile, whereas the 25% Ni sample exhibits a broad peak spanning 411–697 °C, with a shoulder extending to 800 °C, making individual maxima difficult to resolve. As with the as-synthesized catalysts, XRD results (Figure 2) reveal no bulk oxidized nickel phases after DRM, and the tungsten oxide content remains low (up to 2 wt.%). Therefore, the observed hydrogen consumption is attributed to the reduction in surface oxidized nickel and tungsten species formed during DRM. In addition, previous studies [44] on tungsten carbide samples containing free carbon have reported hydrogen consumption peaks in the range of 600–800 °C, associated with CH_4_ formation by hydrogen interaction with carbon. Based on the XRD results of our spent samples (Figure 2), the hydrogen consumption observed between 650 and 800 °C can likewise be linked to CH_4_ formation through hydrogen interaction with graphite produced during DRM. This suggests that the graphitization process is at least partially reversible in this temperature range under hydrogen treatment.

### 3.3. Raman Spectroscopy

**Figure 4:**
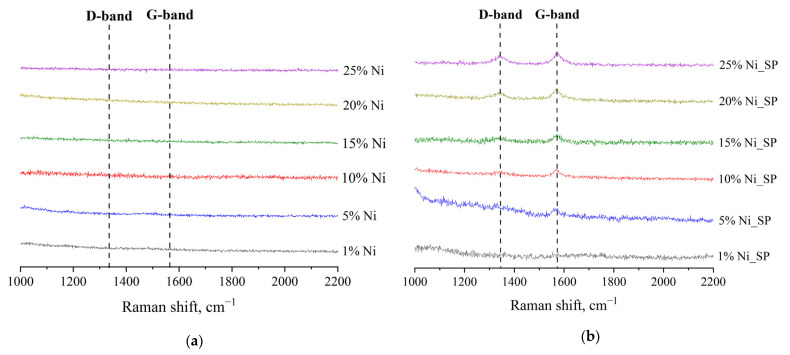
Raman spectra of (**a**) as-synthesized and (**b**) spent catalysts. DRM conditions: T = 800 °C, P = 1 atm, CH_4_:CO_2_ ratio = 1.00, WHSV = 12,000 mL∙g^−1^∙h^−1^.

Further characterization of the as-synthesized and spent catalysts was carried out using Raman spectroscopy (Figure 4) to provide insight into carbon formation and its structural characteristics. In the Raman spectra of the as-synthesized samples (Figure 4a), no bands characteristic of carbonaceous species were detected. In particular, neither the G band (~1580 cm^–1^), associated with sp^2^–hybridized carbon bonds typical of graphitic structures, nor the D band (~1350 cm^–1^), related to structural disorder and amorphous carbon [37,47,48], was observed. This absence confirms that the synthesis and pretreatment steps did not introduce detectable carbon deposits on the catalyst surface.

In contrast, both the G and D bands are clearly visible in the Raman spectra of the spent catalysts after DRM (Figure 4b), indicating carbon deposition during the reaction. The intensity of these bands increases systematically with increasing Ni content, in agreement with the XRD results (Figure 2), suggesting that higher Ni loading promotes carbon formation under the tested conditions. The I_G_/I_D_ ratio, which reflects the degree of graphitization of the carbon formed, reveals that the deposited carbon is not well-ordered. The broadening of both G and D bands, along with the prominent D band, indicates that the carbon formed has a disordered graphitic structure with lattice distortions, stacking faults and partial amorphization. This structural disorder can influence catalyst stability, as less ordered carbon is generally more reactive and can be removed under oxidative or gasifying conditions.

### 3.4. Catalytic Studies

**Figure 5:**
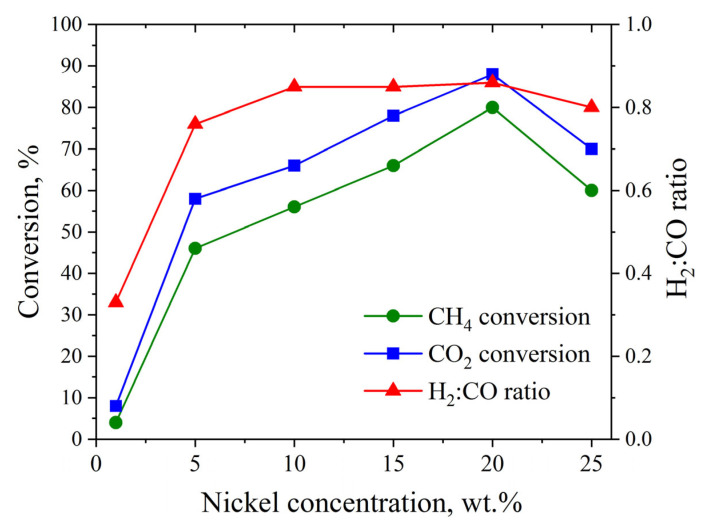
Catalytic performance in DRM (CH_4_ conversion, CO_2_ conversion and H_2_:CO ratio) as a function of nickel concentration (1–25 wt.%) in X%Ni catalysts. DRM conditions: T = 800 °C, P = 1 atm, CH_4_:CO_2_ ratio = 1.00, WHSV = 12,000 mL∙g^−1^∙h^−1^.

Figure 5 shows the catalytic performance in DRM of a series of as-synthesized catalysts with nickel loadings ranging from 1 to 25 wt.%. The results indicate that the conversions of CH_4_ and CO_2_, as well as the H_2_:CO ratio, increase with nickel content, reaching maximum values at 20 wt.% Ni (80% CH_4_ conversion, 88% CO_2_ conversion and H_2_:CO ratio of 0.86), after which all catalytic performance indicators decline. Notably, in all cases, CO_2_ conversion exceeds CH_4_ conversion, and the H_2_:CO ratio remains below 1. This behavior can be attributed to the occurrence of the following side reactions:

1. Reverse Boudouard reaction:

CO_2_ + C → 2CO; (5)

2. Activation of CO_2_ on WC (W_2_C) leading to oxidation of carbides by activated oxygen (O*) [33,37,41,42]:

CO_2_ → CO + O*, (6)

W_2_C + 5O* → 2WO_2_ + CO, (7)

WC+ 3O* → WO_2_ + CO; (8)

3. Reverse water–gas shift (RWGS) reaction:

CO_2_ + H_2_ → CO + H_2_O. (9)

The observed decrease in CH_4_ and CO_2_ conversions for the catalyst with 25 wt.% Ni compared to the 20 wt.% Ni sample is likely due to the following:

1. Boudouard reaction:

2CO → CO_2_ + C; (10)

2. Formation of CH_4_:

C + 2H_2_ ↔ CH_4_. (11)

Both reactions (10) and (11) lead to an increase in the concentration of the initial reagents, thereby effectively reducing their observed conversion. The occurrence of reaction (11) is indirectly supported by the H_2_–TPR (Figure 3b), since, according to the XRD results (Figure 2f), no oxidized phases of nickel and tungsten were detected for the 25 wt.% Ni sample. Such substantial hydrogen consumption cannot be explained only by the reduction in transient surface oxidized species. In addition, the 25 wt.% Ni catalyst exhibits the highest graphite phase content among all studied materials, as confirmed by both XRD (Figure 2f) and Raman spectroscopy (Figure 4b). This suggests that CH_4_ cracking is more intensive in this sample, consistent with reaction (Equation (12)):

CH_4_ ↔ C + 2H_2_. (12)

It can be assumed that a cyclic process takes place: during CH_4_ cracking, carbon and hydrogen are generated, then hydrogen reacts with carbon to regenerate CH_4_, continuing the cycle.

Based on the combined physicochemical and catalytic data, it can be concluded that the optimal nickel content in the catalyst is 20 wt.%. Increasing the nickel concentration to 25 wt.% intensifies CH_4_ cracking, alongside the Boudouard reaction and CH_4_ formation, which together effectively lower the conversion of the initial reactants. Conversely, decreasing the nickel content promotes oxidation of tungsten carbide, as observed for samples with 1, 10 and 15 wt.% Ni (Figure 2a,c,d). Since tungsten oxides are known to be inactive in DRM under the studied conditions, their formation during prolonged catalyst operation can lead to catalyst deactivation. No correlation between catalytic performance and the average nickel crystallite size was observed.

**Figure 6:**
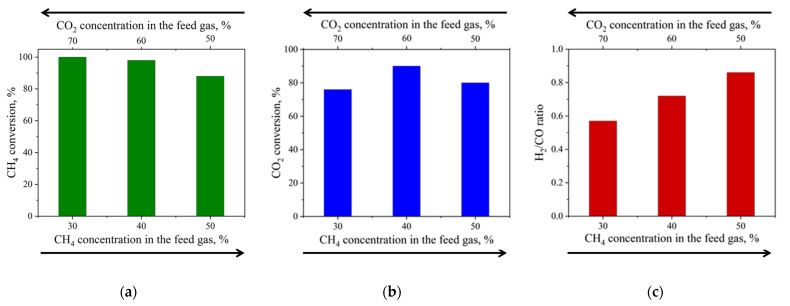
Effect of CH_4_ and CO_2_ concentrations in the feed gas on DRM performance over the 20%Ni catalyst: (**a**) CH_4_ conversion, (**b**) CO_2_ conversion and (**c**) H_2_:CO ratio. DRM conditions: T = 800 °C, P = 1 atm, WHSV = 12,000 mL∙g^−1^∙h^−1^.

Since the optimal nickel content in the catalyst was determined to be 20 wt.% and, as reported in our previous study [29], this sample exhibits a decline in catalytic efficiency during DRM due to the carbon deposition rate exceeding its removal from the catalyst surface, a potential solution is to increase the CO_2_ concentration relative to CH_4_ in the feed. To explore this, we evaluated the influence of the feed composition on the catalytic performance of the 20% Ni sample, with the results shown in Figure 6. The CH_4_ conversion reaches a maximum (of 100%) at a CH_4_:CO_2_ ratio of 0.43 (Figure 6a). Increasing the CH_4_ fraction decreases its conversion to 98% at CH_4_:CO_2_ = 0.67 and further to 88% at CH_4_:CO_2_ = 1.00. In contrast, CO_2_ conversion is highest (90%) at CH_4_:CO_2_ = 0.67, while either increasing or decreasing this ratio lowers CO_2_ conversion (Figure 6b). The H_2_:CO ratio peaks at 0.86 when CH_4_:CO_2_ ratio is 1.00 (Figure 6c). Therefore, using a feed of 40 vol.% CH_4_ and 60 vol.% CO_2_ (CH_4_:CO_2_ = 0.67) enables simultaneous high CH_4_ (98%) and CO_2_ (90%) conversions, albeit with a slightly lower H_2_:CO ratio of 0.72.

**Figure 7:**
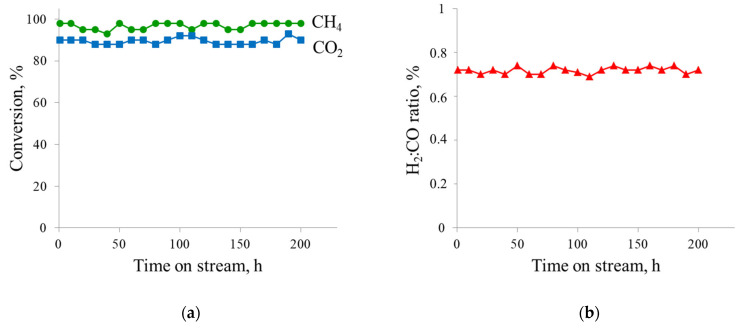
Long-term performance of 20%Ni catalyst in DRM: (**a**) CH_4_ and CO_2_ conversion; (**b**) H_2_:CO ratio vs. time on stream. DRM conditions: T = 800 °C, P = 1 atm, CH_4_:CO_2_ = 0.67 and WHSV = 12,000 mL∙g^−1^∙h^−1^.

To verify the hypothesis that excess CO_2_ helps balance the rates of carbon formation and removal on the catalyst surface, the 20% Ni catalyst was tested in DRM for 200 h at 800 °C, atmospheric pressure and a WHSV of 12,000 mL∙g^−1^∙h^−1^, using a feed containing 40 vol.% CH_4_ and 60 vol.% CO_2_. As shown in Figure 7, no catalyst deactivation was observed during the entire 200 h DRM operation test. The CH_4_ and CO_2_ conversions remained stable at approximately 98% and 90%, respectively, with a constant H_2_:CO ratio of 0.72 throughout the run.

In addition, under these operating conditions, XRD analysis (Figure 2e) shows that after 200 h of DRM, the W_2_C phase is completely transformed into the more stable WC phase. The graphite content is significantly reduced to 1 wt.% compared to 7 wt.% when using a feed with equal CH_4_ and CO_2_ proportions (CH_4_:CO_2_ = 1.00). This confirms that using an excess of CO_2_ relative to CH_4_ in the feed effectively balances the rates of carbon formation and removal from the catalyst surface. Consequently, the oxidation / (re)carbonization cycle (Equations (13)–(18)) is more efficiently sustained, ensuring long-term catalyst stability during DRM.

CH_4_ → C + 2H_2_ (13)

CO_2_ → CO + O* (14)

W_2_C + 5O* → 2WO_2_ + CO (15)

WC + 3O* → WO_2_ + CO (16)

2WO_2_ + 5C* → W_2_C + 4CO (17)

WO_2_ + 3C* → WC + 2CO (18)

It should be emphasized that our 20% Ni catalyst is not inferior, and in some cases even superior, in catalytic efficiency for DRM compared with nickel- and/or cobalt-based tungsten carbide catalysts reported in the literature (Table 2). Moreover, despite the increase in nickel crystallite size during DRM (15 nm for the as-synthesized catalyst to 20 nm after 200 h on stream, Figure 2e), the 20% Ni catalyst exhibits no detectable deactivation under the studied conditions, as measured by catalytic performance, over 200 h of continuous operation.

To place these results in context, a comparison with similar catalysts reported in the literature was conducted. As discussed in our previous work [29], Ni/WC catalysts exhibit competitive activity and selectivity in DRM relative to other non-noble metal catalysts under comparable reaction conditions. The optimized 20% Ni catalyst achieves high CH_4_ conversion and maintains stability over 200 h, surpassing the durability reported for the Ni and/or Co and tungsten carbide catalysts (Table 2), as said above. The Ni/WC catalysts developed in this study thus represent a promising balance between cost-effectiveness, activity and long-term stability, demonstrating their potential for practical DRM applications.

## 4. Conclusions

A series of monometallic nickel catalysts supported on tungsten carbide, with Ni loadings ranging from 1 to 25 wt%, was successfully prepared using the deposition–precipitation method with NaOH. Comprehensive characterization by XRD, H_2_–TPR and Raman spectroscopy revealed phase compositions and structural transformations before and after DRM. Catalytic testing at 800 °C and atmospheric pressure demonstrated that a Ni loading of 20 wt.% provided the optimal balance of activity and stability. Both lower and higher Ni loadings resulted in diminished performance due to structural changes such as tungsten carbide oxidation and increased carbon deposition. Adjusting the CH_4_:CO_2_ feed ratio identified a value of 0.67 (40 vol.% CH_4_ and 60 vol.% CO_2_), which facilitated high CH_4_ and CO_2_ conversions of 98% and 90%, respectively. Under these conditions, the catalyst maintained stable operation for at least 200 h TOS, attributed to an effective oxidation / (re)carbonization cycle that balanced carbon formation and removal. This study provided novel insights into the role of nickel loading and feed composition in tailoring catalyst performance and durability for sustainable DRM processes. Future work will focus on further optimizing the 20% Ni catalyst to achieve a syngas H_2_:CO ratio closer to 1, improving its applicability for downstream chemical synthesis.

## Figures and Tables

**Table 1:** Summary of physico-chemical parameters of 20% Ni/WC_DP catalyst. Data adapted from a previous study [29].

Catalyst	Phase Detected by XRD	w_i_/Σw (%) by XRD	Phase Observed by SAED	SSA (m^2^/g)	Pore Size (nm)	Pore Volume (cm^3^/g)	Average Size of Ni NPs ^b^
20%Ni/WC_DP ^a^	Ni (cubic) WC (hexagonal) W_2_C (hexagonal)	27 63 10	Ni (220) W_2_C (201), (203), (300), (102) WC (100), (101), (111)	38.4	9.9	0.05	15.4

^a^ designated as 20%Ni in current study. ^b^ determined by TEM. w_i_/Σw—obtained phase weight proportions (%). SSA—specific surface area (m^2^/g).

**Table 2:** Catalytic performance of catalysts based on Ni and/or Co and tungsten carbide in DRM.

Catalyst	Reaction Conditions	Conversion, %		H_2_:CO	Ref.
		CH_4_	CO_2_		
20%Ni ^a^	P = 1 atm T = 800 °C CH_4_:CO_2_ = 0.43–1.00 WHSV = 12,000 mL·h^−1^·g_cat_^−1^ TOS = 200 h	88–100	76–90	0.57–0.86	This work
20%Ni/WC_DP ^a^	P = 1 atm T = 800 °C CH_4_:CO_2_ = 1 WHSV = 3600–12,000 mL·h^−1^·g_cat_^−1^ TOS = 80 h	80–96	88–94	0.79–1.00	[29]
Ni–WC_x_	P = 1 atm T = 800 °C CH_4_:CO_2_ = 1 WHSV = 36,000 mL·h^−1^·g_cat_^−1^ TOS = 25 h	45	48	0.6	[30]
Co_6_W_6_C	P = 5 atm T = 850 °C CH_4_:CO_2_ = 1 WHSV = 11,200 scc·h^−1^·g_cat_^−1^ TOS = 100 h	82	78	1.00	[31]
Ni–WC	P = 1 atm T = 800 °C CH_4_:CO_2_ = 0.67–1.5 Flow = 50 mL·min^−1^ TOS = 20 h	60–98	75–85	0.70–0.80	[33]
Co/WC–AC	P = 1 atm T = 800 °C CH_4_:CO_2_ = 1 Flow = 120 mL·min^−1^ TOS = 24 h	86	86	0.90	[34]
Ni-Co/WC–AC	P = 1 atm T = 800 °C CH_4_:CO_2_ = 1 Flow = 120 mL·min^−1^ TOS = 24 h	92	91	0.98	[34]
Co/WC–AC	P = 1 atm T = 800 °C CH_4_:CO_2_ = 1 GHSV = 2400 mL·h^−1^·g_cat_^−1^ Flow = 100 mL·min^−1^ TOS = 12 h	95	95	0.92	[35]
Ce–Co/WC–AC	P = 1 atm T = 800 °C CH_4_:CO_2_ = 1 Flow =120 mL·min^−1^ TOS = 10 h	91	93	0.92	[36]
Ni–WC	P = 1 atm T = 800 °C CH_4_:CO_2_ = 1 WHSV = 6000 scc·h^−1^·g_cat_^−1^ Flow = 120 mL·min^−1^ TOS = 12 h	82	90	n.d. ^b^	[37]
Y–Co/WC–AC	P = 1 atm T = 800 °C CH_4_:CO_2_ = 1 GHSV = 4800 mL·h^−1^·g_cat_^−1^ Flow =120 mL·min^−1^ TOS = 10 h	92	94	0.85	[38]
Co–WC	P = 3.4 atm T = 850 °C CH_4_:CO_2_ = 1 WHSV = 9000 scc·h^−1^·g_cat_^−1^ TOS = 100 h	78	70	1.65	[39]
Ni–WC	P = 3.4 atm T = 850 °C CH_4_:CO_2_ = 1 WHSV = 9000 scc·h^−1^·g_cat_^−1^ TOS = 100 h	85	78	1.62	[39]
Ni–WC	T = 800 °C CH_4_:CO_2_ = 1 GHSV = 5000 mL·h^−1^·g_cat_^−1^ TOS = 16 h	75	50	85	[40]
Ni_17_W_3_/SiO_2_	P = 1 atm T = 800 °C CH_4_:CO_2_ = 1 WHSV = 96,000 mL·h^−1^·g_cat_^−1^ TOS = 30 h	60	70	n.d. ^b^	[41]
Ni–WC_x_ nanospheres	P = 1 atm T = 800 °C CH_4_:CO_2_ = 1 WHSV = 18,000 mL·h^−1^·g_cat_^−1^ TOS = 29 h	68	80	n.d. ^b^	[42]

^a^ the same catalyst. ^b^ no data.

## Data Availability

The original contributions presented in this study are included in the article. Further inquiries can be directed to the corresponding authors.

## References

[1] Norhasyima R.S., Mahlia T.M.I. (2018). Advances in CO_2_ Utilization Technology: A Patent Landscape Review. J. CO2 Util..

[2] Maestri M., Vlachos D.G., Beretta A., Groppi G., Tronconi E. (2009). A C1 Microkinetic Model for Methane Conversion to Syngas on Rh/Al_2_O_3_. AIChE J..

[3] Naidja A., Krishna C.R., Butcher T., Mahajan D. (2003). Cool Flame Partial Oxidation and Its Role in Combustion and Reforming of Fuels for Fuel Cell Systems. Prog. Energy Combust. Sci..

[4] Fan M., Abdullah A.Z., Bhatia S. (2009). Catalytic Technology for Carbon Dioxide Reforming of Methane to Synthesis Gas. ChemCatChem.

[5] Nedolivko V.V., Zasypalov G.O., Vutolkina A.V., Gushchin P.A., Vinokurov V.A., Kulikov L.A., Egazar’yants S.V., Karakhanov E.A., Maksimov A.L., Glotov A.P. (2020). Carbon Dioxide Reforming of Methane. Russ. J. Appl. Chem..

[6] Er-Rbib H., Bouallou C., Werkoff F. (2012). Dry Reforming of Methane–Review of Feasibility Studies. Chem. Eng..

[7] Arora S., Prasad R. (2016). An Overview on Dry Reforming of Methane: Strategies to Reduce Carbonaceous Deactivation of Catalysts. RSC Adv..

[8] Pakhare D., Spivey J. (2014). A Review of Dry (CO_2_) Reforming of Methane over Noble Metal Catalysts. Chem. Soc. Rev..

[9] Shafiee P., Alavi S.M., Rezaei M. (2021). Mechanochemical Synthesis Method for the Preparation of Mesoporous Ni–Al_2_O_3_ Catalysts for Hydrogen Purification via CO_2_ Methanation. J. Energy Inst..

[10] Budiman A.W., Song S.-H., Chang T.-S., Shin C.-H., Choi M.-J. (2012). Dry Reforming of Methane over Cobalt Catalysts: A Literature Review of Catalyst Development. Catal. Surv. Asia.

[11] Sharifianjazi F., Esmaeilkhanian A., Bazli L., Eskandarinezhad S., Khaksar S., Shafiee P., Yusuf M., Abdullah B., Salahshour P., Sadeghi F. (2022). A Review on Recent Advances in Dry Reforming of Methane over Ni-and Co-Based Nanocatalysts. Int. J. Hydrogen Energy.

[12] Gonzalez-delaCruz V.M., Pereñiguez R., Ternero F., Holgado J.P., Caballero A. (2012). In Situ XAS Study of Synergic Effects on Ni–Co/ZrO_2_ Methane Reforming Catalysts. J. Phys. Chem. C.

[13] Salaev M.A., Liotta L.F., Vodyankina O.V. (2022). Lanthanoid-Containing Ni-Based Catalysts for Dry Reforming of Methane: A Review. Int. J. Hydrogen Energy.

[14] Li M., Sun Z., Hu Y.H. (2021). Catalysts for CO_2_ Reforming of CH_4_: A Review. J. Mater. Chem. A.

[15] Owgi A.H.K., Jalil A.A., Hussain I., Hassan N.S., Hambali H.U., Siang T.J., Vo D.V.N. (2021). Catalytic Systems for Enhanced Carbon Dioxide Reforming of Methane: A Review. Environ. Chem. Lett..

[16] Nguyen D.L.T., Tran A.V., Vo D.-V.N., Nguyen H.T., Rajamohan N., Trinh T.H., Nguyen T.L., Le Q.V., Nguyen T.M. (2024). Methane Dry Reforming: A Catalyst Challenge Awaits. J. Ind. Eng. Chem..

[17] Ighalo J.O., Amama P.B. (2024). Recent Progress in the Design of Dry Reforming Catalysts Supported on Low-Dimensional Materials. J. CO_2_ Util..

[18] Guil-López R., Nieto E., Botas J.A., Fierro J.L.G. (2012). On the Genesis of Molybdenum Carbide Phases during Reduction-Carburization Reactions. J. Solid State Chem..

[19] Levy R.B., Boudart M. (1973). Platinum-like Behavior of Tungsten Carbide in Surface Catalysis. Science.

[20] York A.E., Claridge J., Brungs A., Tsang S., Green M.H. (1997). Molybdenum and Tungsten Carbides as Catalysts for the Conversion of Methane to Synthesis Gas Using Stoichiometric Feedstocks. Chem. Commun..

[21] Yan Q., Lu Y., To F., Li Y., Yu F. (2015). Synthesis of Tungsten Carbide Nanoparticles in Biochar Matrix as a Catalyst for Dry Reforming of Methane to Syngas. Catal. Sci. Technol..

[22] Gavrilova N.N., Sapunov V.N., Skudin V.V. (2019). Intensification of Dry Reforming of Methane on Membrane Catalyst. Chem. Eng. J..

[23] Grigoryan R.R., Aloyan S.G., Harutyunyan V.R., Arsentev S.D., Tavadyan L.A. (2019). Dry Reforming of Methane over Nanosized Tungsten Carbide Powders Obtained by Mechanochemical and Plasma-Mechanochemical Methods. Pet. Chem..

[24] Zhang Q., Pastor-Pérez L., Gu S., Ramirez Reina T. (2020). Transition Metal Carbides (TMCS) Catalysts for Gas Phase CO_2_ Upgrading Reactions: A Comprehensive Overview. Catalysts.

[25] Czaplicka N., Rogala A., Wysocka I. (2021). Metal (Mo, W, Ti) Carbide Catalysts: Synthesis and Application as Alternative Catalysts for Dry Reforming of Hydrocarbons—A Review. Int. J. Mol. Sci..

[26] Vasilevich A.V., Baklanova O.N., Lavrenov A.V. (2020). Molybdenum Carbides: Synthesis and Application in Catalysis. Solid Fuel Chem..

[27] Abdullah M., Sajjadi B. (2024). Sustainable Conversion of Natural Gas to Hydrogen Using Transition Metal Carbides. Int. J. Hydrogen Energy.

[28] LaMont D.C., Thomson W.J. (2005). Dry Reforming Kinetics over a Bulk Molybdenum Carbide Catalyst. Chem. Eng. Sci..

[29] Bolatova Z., German D., Pakrieva E., Pak A., Larionov K., Carabineiro S.A.C., Bogdanchikova N., Kolobova E., Pestryakov A. (2022). Ni, Co and Ni-Co-Modified Tungsten Carbides Obtained by an Electric Arc Method as Dry Reforming Catalysts. Catalysts.

[30] Zhang Y., Zhang S., Zhang X., Qiu J., Yu L., Shi C. (2015). Ni Modified WCx Catalysts for Methane Dry Reforming. Advances in CO_2_ Capture, Sequestration, and Conversion.

[31] Iyer M.V., Norcio L.P., Punnoose A., Kugler E.L., Seehra M.S., Dadyburjor D.B. (2004). Catalysis for Synthesis Gas Formation from Reforming of Methane. Top. Catal..

[32] Shao H., Kugler E.L., Dadyburjor D.B., Rykov S.A., Chen J.G. (2009). Correlating NEXAFS Characterization of Co–W and Ni–W Bimetallic Carbide Catalysts with Reactivity for Dry Reforming of Methane. Appl. Catal. A Gen..

[33] Barbosa R.D., Baldanza M.A.S., de Resende N.S., Passos F.B., da Silva V.L.D.S.T. (2021). Nickel–Promoted Molybdenum or Tungsten Carbides as Catalysts in Dry Reforming of Methane: Effects of Variation in CH_4_/CO_2_ Molar Ratio. Catal. Lett..

[34] Zhang X., Wang J., Zhang G., Liu J., Wang Y., Zhao Y., Li G. (2023). Co–Ni/WC-AC catalysts for dry reforming of methane: The role of Ni species. Int. J. Hydrogen Energy.

[35] Li S., Zhang G., Wang J., Liu J., Lv Y. (2021). Highly stable activity of cobalt based catalysts with tungsten on Tungsten Carbide-Activated Carbon for CO_2_ Reforming of CH_4_ to Produce Syngas. Int. J. Hydrogen Energy.

[36] Li S., Wang J., Zhang G., Liu J., Lv Y., Zhang Y. (2022). Highly active and stable cobalt catalysts with a tungsten carbide-activated Carbide-Activated Carbon Support for Dry Reforming of Methane: Role of Tungsten Carbide. Fuel.

[37] Yao Z., Jiang J., Zhao Y., Luan F., Zhu J., Shi Y., Gao H., Wang H. (2016). Insights into the deactivation mechanism of metal carbide catalysts for dry reforming of methane via comparison of nickel-modified molybdenum and tungsten carbides. RSC Adv..

[38] Zhang Y., Liang Z., Zhang G., Liu J., Wang Y., Zhao Y., Li G., Lv Y. (2022). Highly Active and Stable Cobalt Catalysts with a Tungsten Carbide-Activated Carbon Support for Dry Reforming of Methane: Effect of the Different Promoters. Catal. Sci. Technol..

[39] Shao H. (2006). Bimetallic Carbides as Catalysts for Dry Reforming and Steam Reforming. Ph.D. Thesis.

[40] Wysocka I., Czaplicka N., Pawelczyk E., Karczewski J., Sobczak J., Bielan Z., Maciejewski M., Kościelska B., Rogala A. (2023). Novel Sugar-Based Nickel-Tungsten Carbide Catalysts for Dry Reforming of Hydrocarbons. J. Ind. Eng. Chem..

[41] Zhang S., Shi C., Chen B., Zhang Y., Qiu J. (2015). An Active and Coke-Resistant Dry Reforming Catalyst Comprising Nickel–Tungsten Alloy Nanoparticles. Catal. Commun..

[42] Liang N., Xing J. (2018). Study on Catalytic Roles of Tungsten Carbide Nanospheres for CH_4_/CO_2_ Reforming Over Ni Based Catalysts. Top. Chem. Mater. Eng..

[43] Pak A.Y., Kokorina A.I. (2021). Effect of Energy on the Phase Composition of the Product of Arc Discharge Synthesis in the Tungsten–Carbon System Obtained in a Self-Shielding Autonomous Gas Environment. Inorg. Mater. Appl. Res..

[44] Okamoto H., Kawamura G., Ishikawa A., Kudo T. (1987). Characterization of Oxygen in WC Catalysts and Its Role in Electrocatalytic Activity for Methanol Oxidation. J. Electrochem. Soc..

[45] Xie Z., Li M., Zhou Y., Feng Y., Song X., Li L., Ding W., Wei Z. (2024). Temperature-Induced Interface Hybridization of WC Boosts NiCu Activity for Alkaline Hydrogen Oxidation Reaction. Small Methods.

[46] Zhou K., Du X., Zhou L., Yang H., Lei X., Zeng Y., Li D., Hu C. (2021). The Deoxygenation of Jatropha Oil to High Quality Fuel via the Synergistic Catalytic Effect of Ni, W_2_C and WC Species. Catalysts.

[47] Shishanov M.V., Luchkin M.S., Ivanova A.N., Morozov A.A. (2024). Raman Spectroscopy as Method for the Analysis of Carbon Materials. Coke Chem..

[48] Pawelczyk E., Wysocka I., Dymerski T., Gębicki J. (2024). Catalytic Activity of Ni-MgAl_2_O_4_ Modified with Transition Metal (Ti, Mo, W) Carbides as Potential Catalysts for Resource Recovery via Dry Reforming of Waste Plastics. Catal. Today.

